# 肺癌外周免疫评分的最新研究进展

**DOI:** 10.3779/j.issn.1009-3419.2025.102.21

**Published:** 2025-05-20

**Authors:** Fan XU, Bin LUO, Jianhui TIAN, Yun YANG, Zhenyang CHENG, Youjun LIU

**Affiliations:** ^1^200071 上海，上海中医药大学附属市中医医院肿瘤临床医学中心; ^1^Department of Oncology,Shanghai University of Traditional Chinese Medicine, Shanghai 200071, China; ^2^200071 上海，上海中医药大学附属市中医医院肿瘤研究所; ^2^Research Center for Cancer, Shanghai Municipal Hospital of Traditional Chinese Medicine,Shanghai University of Traditional Chinese Medicine, Shanghai 200071, China

**Keywords:** 肺肿瘤, 免疫治疗, 外周免疫评分, 预测模型, Lung neoplasms, Immunotherapy, Peripheral immunoscore, Predictive models

## Abstract

肺癌是发病率和死亡率最高的恶性肿瘤，肿瘤原发灶-淋巴结-转移（tumor-node metastasis, TNM）分期在肺癌相关预测中逐渐显现局限性，亟待构建新型临床预测模型指导肺癌防治。近年来，外周免疫评分作为一种基于外周免疫相关参数的综合评估体系，在预测模型构建中的价值逐渐凸显。该评分通过量化外周免疫成分的数量及比例，可动态反映机体整体免疫功能状态及肿瘤微环境特征。本文系统总结了外周免疫评分在肺癌早期诊断、用药疗效预测、不良反应预警及预后评估中的最新研究进展，旨在挖掘其潜在的临床应用价值，为开发新型肺癌相关预测模型提供一定的思路与方向。

## Abstract (en)

Lung cancer is the malignant tumor with the highest morbidity and mortality. The tumor-node-metastasis (TNM) staging has gradually shown its limitations in the accurate prediction of lung cancer, so it is urgent to construct a new clinical predictive model to guide the prevention and treatment of lung cancer. In recent years, as a comprehensive evaluation system based on peripheral immune related parameters, the value of peripheral immunoscore in the construction of predictive model has gradually become prominent. By quantifying the quantity and proportion of immune components in peripheral blood, the score can dynamically reflect the overall immune function and tumor microenvironment characteristics of the body. This paper systematically summarizes the latest research progress of peripheral immunoscore in early diagnosis, drug efficacy prediction, early warning of adverse reactions and prognosis evaluation of lung cancer, aiming to tap its potential clinical application value and provide some ideas and directions for developing new lung cancer-related predictive models.

肺癌是全球发病率与死亡率最高的恶性肿瘤^[1]^，5年生存率仍不足15%^[2]^。以手术、放化疗、靶向及免疫治疗为核心的肺癌综合防治体系仍待完善，而构建相关预测模型是肺癌临床防治的首要迫切需求。肿瘤原发灶-淋巴结-转移（tumor-node-metastasis, TNM）分期是目前国际上最为通用的肺癌相关预测模型。然而，随着对肿瘤生物学机制的不断探索与免疫治疗等新型策略的不断开发，仅采用TNM分期来指导现有临床防治已变得尤为局限^[3,4]^。

随着肺癌免疫治疗的不断获益，以机体免疫状态评估为核心的预测模型构建成为热点^[5,6]^。免疫评分是基于免疫相关参数而建立的综合评分体系，以评估机体免疫功能状态，此类肺癌相关预测模型具有潜在的临床价值。目前，基于肿瘤微环境的免疫评分日趋成熟，并实现有效临床转化^[7-10]^。但也面临着一系列挑战，如肿瘤异质性、创伤性大、不可重复取样、无法实时动态监测等。在此背景下，“外周免疫评分”概念被提出。外周免疫评分基于外周血免疫相关参数，相比较而言，其可有效避免肿瘤异质性带来的预测误差，更能反映机体整体免疫状态和肿瘤微环境免疫浸润水平，且具有采样方便、可重复性、微创性、实时动态监测等优势^[11]^。目前外周免疫评分研究尚在起步阶段，其在肺癌相关预测模型构建中有着巨大的应用前景，值得深入探索。

## 1 外周免疫评分概念的提出和优势分析

2006年，Galon等^[12]^首次提出“免疫结构”概念，即肿瘤组织不同区域内的获得性免疫细胞类型、功能、定位、密度和位置。并在2009年通过大规模队列研究，成功构建了首个基于肿瘤浸润淋巴细胞的免疫相关预测模型，以预测结直肠癌患者的预后^[13]^。2012年，Galon等^[14]^基于“免疫结构”进一步提出“免疫评分”，即通过量化肿瘤组织区域内免疫相关参数而得出的综合评分，并率先在结肠癌中构建了免疫评分，用于结肠癌的复发风险预测，受到领域内的广泛认可。至2020年，欧洲肿瘤内科学会指南将免疫评分与TNM结合，形成TNM-I分期，推广至临床II-III期结肠癌患者的预后预测中。此后，免疫评分逐渐成熟，现已拓展至肺癌、膀胱癌、肝癌等其他癌种^[15]^。然而，这类基于肿瘤微环境的免疫评分在实际应用中仍面临诸多瓶颈，如肿瘤异质性与复杂的肿瘤微环境，单一样本无法准确反映肿瘤微环境免疫浸润情况，且创伤性大、不可重复取样、无法实时动态监测等，为构建精准、高效的临床预测模型带来一定的困难与挑战。

近年来，随着流式细胞术、酶联免疫吸附试验、多重荧光素酶标法等检测技术的不断发展，基于外周免疫相关参数的“外周免疫评分”作为一个新兴概念被提出。外周免疫评分是一种通过量化外周血中免疫相关参数（包括免疫细胞、可溶性因子、循环生物标志物及近年来发现的新兴指标），来综合评估机体整体免疫功能状态和肿瘤微环境免疫浸润水平的一种动态评分体系。与基于肿瘤微环境的免疫评分相比，外周免疫评分可避免肿瘤异质性与肿瘤微环境的复杂性等带来的干扰，更能反映机体整体抗肿瘤免疫应答强度及免疫抑制水平。同时，外周血获取方便、重复性强、创伤性小，可实时动态监测，在实现肿瘤精准预测中展现出巨大的临床价值^[16-18]^。

## 2 外周免疫评分在肺癌相关预测中的应用

### 2.1 协助肺癌的早期诊断

肺结节具有一定的癌变风险，然而影像学表现往往缺乏特异性，容易导致漏诊或过度治疗，迫切需要开发更为精准、无创的技术以评估其癌变风险。研究^[19]^表明，外周免疫功能状态与肺结节癌变风险具有显著相关性。Yang等^[20]^以外周血T细胞受体（T-cell receptor, TCR）库为核心参数构建了相关外周免疫评分——“Lung Cancer Prediction”（LCP）。该研究共收集100例I期肺癌患者和99例良性肺结节患者的外周血样本，通过高通量测序技术进行外周血TCR库分析，筛选出15个具有特征性的TCR克隆作为自变量（5个良性特征性TCR克隆和10个恶性特征性TCR克隆）。基于这15个特征性TCR克隆参数，利用反向传播神经网络模型构建了肺结节癌变风险评分体系LCP。结果显示，LCP验证集的受试者工作特征曲线下面积（area under the curve, AUC）、阳性预测值（positive predictive value, PPV）和阴性预测值（negative predictive value, NPV）分别达到0.74、80%和71.4%，证实LCP具有良好的预测价值，可用于肺结节癌变风险的早期评估。

肺癌分为原发性肺癌和转移性肺癌，肿瘤来源鉴定对于临床用药决策至关重要。基于此，Ganesh等^[21]^开发了一种可协助诊断原发性/转移性肺癌的外周免疫评分。研究收集了健康对照组、原发性肺癌组和转移性肺癌组的外周血样本，首先利用团队自主研发的超灵敏纳米传感器T-sense，在单细胞水平上建立了一套高度敏感的外周血T细胞特征集。然后，基于这套特征集参数，通过机器学习算法进行特征选择和降维处理，构建了可用于鉴别原发性肺癌和转移性肺癌的外周免疫评分体系。模型评估结果显示，该评分体系对于原发性肺癌的预测特异性和敏感性分别达到了94.1%和100%，对转移性肺癌的预测特异性和敏感性则分别达到了97.9%和94.4%，展现出良好的预测性能，为协助临床肺癌来源的精准鉴定提供了更多的选择。

### 2.2 用药疗效预测，筛选获益人群

近年来，以免疫检查点抑制剂（immune checkpoint inhibitors, ICIs）、嵌合抗原受体T细胞（chimeric antigen receptor T-cell, CAR-T）疗法等为代表的新兴免疫治疗在适宜癌种中取得显著突破，肿瘤防治理念已从“以瘤为主”向“以人为本，人瘤并重”转变。然而，ICIs响应率低，仅部分人群获益，亟待构建相关预测模型指导优势人群筛选。面对这一临床难题，Wei等^[22]^以外周血细胞因子谱为核心参数，融入机器学习算法构建了一种新型外周免疫评分，用于精准预测非小细胞肺癌（non-small cell lung cancer, NSCLC）患者对ICIs的响应率。该项临床试验共纳入222例接受程序性死亡受体1/程序性死亡配体1（programmed death receptor-1/programmed death ligand 1, PD-1/PD-L1）单抗治疗或联合化疗的NSCLC患者。治疗前和治疗后6周分别检测外周血93种细胞因子的血浆浓度，通过随机生存森林分析，筛选得到特征性细胞因子，构建外周免疫评分，来预测接受ICIs治疗的响应率及总生存期（overall survival, OS）。结果显示，在群体水平上，两组验证队列的一致性指数分别达到了0.700和0.751，表明所构建的外周免疫评分能够较准确地识别响应率低以及具有较差OS的NSCLC患者，预测性能良好。在此基础上，研究进一步纳入了其他外周血因素及患者一般临床特征，结果证实预测模型的精准度得到了进一步的提升。

SAKK 16/14研究^[23]^报道，围术期联合度伐利尤单抗和新辅助化疗的患者群体中，达到主要病理反应（main pathological response, MPR）的患者比未达到MPR的患者显示出更长的无进展生存期（progression-free survival, PFS）和更高的OS率。然而，只有一小部分NSCLC患者在接受上述治疗时可获得高MPR。明确MPR相关参数、构建相关预测模型对于筛选高MPR人群至关重要^[24-26]^。Peng等^[27]^证实外周血免疫细胞亚群与高MPR显著相关，并筛选得到3类特征性免疫细胞亚群：CD3^+^CD56^+^自然杀伤T（natural killer T, NKT）细胞、CD3^-^CD56^+^ NK和CD4^+^CD45RA^-^ T细胞，可作为高MPR的独立预测因素。在此基础上，Peng等^[27]^基于支持向量机（support vector machine, SVM）构建了外周免疫评分——LIP-SVM。结果显示，LIP-SVM的内部验证集和外部验证集均展现出高水平的预测能力，显著高于年龄、组织学、放射反应等其他变量的预测性能。

另外，外周免疫评分亦可用于肺癌靶向治疗的相关预测。如Chen等^[28]^设计构建了一种以外周血炎性指标为核心的外周免疫评分，称为泛免疫炎症值（pan-immune-inflammation value, PIV），用于临床预测接受一线间变性淋巴瘤激酶（anaplastic lymphoma kinase, ALK）抑制剂治疗的ALK阳性NSCLC患者疗效。该研究共纳入94例接受一线ALK抑制剂的晚期ALK阳性NSCLC患者，检测外周血中中性粒细胞、单核细胞、血小板计数和淋巴细胞计数，以外周血中性粒细胞、单核细胞和血小板计数的乘积再除以淋巴细胞计数，得到每例患者的总评分，然后通过Kaplan-Meier法和*Cox*风险回归来进行相关性分析和生存分析。结果证实，在前期筛选的几个自变量中，仅PIV是结局的独立预测因素（HR=4.70, 95%CI: 2.00-11.02, P<0.001）。PIV是唯一一个可作为接受一线ALK抑制剂的ALK阳性晚期NSCLC患者的PFS和OS的预测评分。

### 2.3 用药后不良反应预测，规避用药风险

免疫相关不良反应（immune-related adverse events, irAEs）是ICIs治疗的常见不良反应，部分反应轻微且可逆，而部分则可能危及生命，需紧急处理^[29]^。针对这一临床迫切需求，Pavan等^[30]^基于外周血炎性标志物构建了可预测临床irAEs的外周免疫评分体系。该研究共纳入184例晚期NSCLC患者，其中142例已接受ICIs治疗，26例尚未接受ICIs治疗。以中性粒细胞与淋巴细胞之比（neutrophil-to-lymphocyte ratio, NLR）和血小板与淋巴细胞之比（platelet lymphocyte ratio, PLR）构建预测模型。多因素分析显示，低NLR、低PLR与irAEs的发生显著相关，是irAEs的独立预测因素（OR=2.3, P=0.020），基于NLR和PLR构建的外周免疫评分可有效预测接受irAEs风险。Lin等^[31]^也展开相应回顾性研究，纳入了138例接受ICIs治疗的晚期NSCLC患者，收集了外周血中免疫、炎性及营养指标，通过LASSO回归结合多变量筛选，得到独立预测变量，构建了可预测严重irAEs的外周免疫评分——AdNLA，验证集的AUC为0.762（95%CI: 0.670-0.854），证实其在严重irAEs的预测上具有较好的应用前景。

此外，有团队将外周免疫指标与其他irAEs预测变量相结合，以进一步提高irAEs风险预测的精准性。如Gao等^[32]^在以NLR、血小板和淋巴细胞作为特征变量的基础上，进一步加入人口统计学、临床病理学和治疗信息，采用多变量Logistic回归构建了基于美国东部肿瘤协作组（Eastern Cooperative Oncology Group, ECOG）体能状态、NLR、血小板和淋巴细胞的irAEs外周免疫评分。结果证实，该外周免疫评分在评估irAEs类型、发病时间、级别和治疗上具有更佳的预测性能（AUC=0.851），为临床医生筛选ICIs治疗人群、制定个体化治疗方案提供了精准、可靠的依据。

### 2.4 协助肺癌的预后评估

复发转移是导致肺癌死亡的主要原因。外周血CD3^+^、CD4^+^、CD8^+ ^T细胞、NK细胞、髓源性抑制细胞、调节性T细胞等免疫参数是肺癌复发或转移的独立预测因素。基于这些独立预测因素构建外周免疫评分，在肺癌复发转移风险预测中具有潜在的应用价值。如Xie等^[33]^构建了一种可用于NSCLC复发转移风险预测的外周免疫评分。该研究收录了7158例NSCLC患者治疗前的外周血NLR、单核细胞计数和血红蛋白等数据，通过多元*Cox*比例风险模型，多因素综合分析得出基于外周血NLR、单核细胞计数和血红蛋白水平的预测模型具有良好的风险预测性能，验证集C-index达0.81。Wang等^[34]^基于72例晚期肺癌患者治疗前1-4个周期循环T细胞受体β链（T cell receptor β, TCRβ）的相关数据，建立了预测晚期肺癌预后的外周免疫评分，对于晚期肺癌的OS具有良好的预测价值。

## 3 人工智能融入外周免疫评分的构建

传统的回归模型，适合较少参数型免疫评分体系的构建，如NLR、PLR、CD4/CD8等，操作简便、成本低，但在预测的灵活性、准确性以及适用范围等方面较为局限。近年来，人工智能（artificial intelligence, AI）技术已深度融入到医学领域中。与传统数据分析方法相比，AI能通过自动特征选择、多模态数据融合、异质性数据适应或正则化技术，更快捷地处理、分析高维参数，发现传统分析方法难以发现的特征与规律，减少信息丢失。基于AI构建的多参数整合模型能够考虑更多的影响因素，捕捉数据之间的复杂关系，减少单一参数带来的偏差和方差，显著提升外周免疫评分的精准性和预测能力^[35-37]^。

本文总结了近年来应用于肿瘤预测模型构建的AI技术：（1）影像组学：影像组学技术通过先进的影像技术及机器学习算法，对肿瘤影像图片进行精确分割、特征提取，并将这些特征进行量化分析，进而构建分类模型，实现基于影像学的预测评估。这一技术能够挖掘影像数据中的深层信息，为肿瘤的早期诊断、疗效评估和预后预测提供有力支持；（2）机器学习与深度学习：机器学习尤其是深度学习，如卷积神经网络、循环神经网络、softmax回归，能够一对一地进行训练，提取与终点任务（如分割、分类和生存预测）有关的特征，实现精准预测。这些技术能够处理大规模、高维度的肿瘤数据，挖掘其中的复杂模式，为肿瘤的个性化治疗提供科学依据；（3）单细胞RNA-seq和空间转录组学结合AI：如spSeudoMap，具有提高分辨率、去卷积和映射、细胞间通讯分析等优势，通过评估肿瘤组织染色图像，可预测患者肿瘤的侵袭性，确定肿瘤细胞的基因组成，以此评估患者预后；（4）人工神经网络、支持向量机、聚类算法等：这些算法可以应用于分析肿瘤基因表达数据，挖掘其中蕴含的知识和规律，建立预测模型。这些算法可应用于分析肿瘤基因表达数据，挖掘其中蕴含的知识和规律，建立预测模型。AI在构建更全面的评估预测模型中具有广阔的前景。因此，将AI融入肺癌外周免疫评分及相关预测模型的构建，可显著提高其精准预测。

## 4 困难与挑战

目前外周免疫评分研究尚在初期阶段，其临床转化仍面临着诸多挑战。首先，检测技术的优化是提升外周免疫评分性能的关键。随着流式细胞术、高通量测序等技术的不断进步，外周免疫评分的检测精度和效率将得到进一步提升。这将使得外周免疫评分能够更加准确、快速地反映患者的免疫状态，为临床预测提供更加可靠的数据支持。其次，免疫相关指标的拓展也是外周免疫评分发展的重要方向。目前外周免疫相关标志物主要涉及免疫细胞分群、可溶性因子，甚至一些已涉及到循环生物标志物、TCR库等免疫参数，未来可以进一步拓展其他与免疫相关的指标，更全面地了解患者的免疫状态，为肺癌预测评估提供更加丰富的信息。此外，多学科合作对于外周免疫评分的研究和应用至关重要。外周免疫评分的研究和应用仍需要多学科支持，未来可以加强肿瘤学、免疫学、生物信息学等领域的交叉，共同推动外周免疫评分在肺癌预测模型构建中的发展。最后，随着外周免疫评分在临床上的广泛应用，标准化与规范化成为亟待解决的问题。需要建立更加标准化和规范化的检测流程和评估标准，确保预测结果的精准性和可比性。这些将为外周免疫评分实现有效临床转化提供更加可靠的保障，大力推动其在肺癌预测领域发挥更大的作用。

## 5 总结与展望

基于“以人为本”的核心理念，外周免疫评分通过微创、动态反映机体整体免疫功能状态和肿瘤微环境免疫浸润水平，为肺癌相关预测模型的构建指引了新的方向。目前外周免疫评分研究已兴起，但仍需要进一步深入探索。未来还需通过多组学整合、AI优化及标准化流程建立，进一步推动外周免疫评分向临床实践转化，最终改善肺癌患者生存结局。
